# Protease Activated Receptors: A Pathway to Boosting Mesenchymal Stromal Cell Therapeutic Efficacy in Acute Respiratory Distress Syndrome?

**DOI:** 10.3390/ijms23031277

**Published:** 2022-01-24

**Authors:** Naveen Gupta

**Affiliations:** Department of Medicine, University of California-San Diego, 9300 Campus Point Drive, #7381, La Jolla, San Diego, CA 92037, USA; n6gupta@health.ucsd.edu

**Keywords:** mesenchymal stromal cells, protease activated receptors, sepsis, lung injury

## Abstract

Acute Respiratory Distress Syndrome is the most common cause of respiratory failure among critically ill patients, and its importance has been heightened during the COVID-19 pandemic. Even with the best supportive care, the mortality rate in the most severe cases is 40–50%, and the only pharmacological agent shown to be of possible benefit has been steroids. Mesenchymal stromal cells (MSCs) have been tested in several pre-clinical models of lung injury and been found to have significant therapeutic benefit related to: (a) potent immunomodulation; (b) secretion of epithelial and endothelial growth factors; and (c) augmentation of host defense to infection. Initial translational efforts have shown signs of promise, but the results have not yielded the anticipated outcomes. One potential reason is the relatively low survival of MSCs in inflammatory conditions as shown in several studies. Therefore, strategies to boost the survival of MSCs are needed to enhance their therapeutic effect. Protease-activated receptors (PARs) may represent one such possibility as they are G-protein coupled receptors expressed by MSCs and control several facets of cell behavior. This review summarizes some of the existing literature about PARs and MSCs and presents possible future areas of investigation in order to develop potential, PAR-modified MSCs with enhanced therapeutic efficiency.

## 1. Introduction

Acute Respiratory Distress Syndrome (ARDS) is the most common cause of acute respiratory failure among critically ill patients. Infections due to bacterial and viral pneumonia as well as severe sepsis from non-pulmonary sources are the most common causes of ARDS. The mortality of moderate to severe ARDS remains high at 30–50% despite optimal supportive care, and during the ongoing SARS-CoV-2 pandemic the mortality rate in the most severe cases has been around 60% [1,2]. Treatment of ARDS is primarily centered on supportive care including low-tidal volume ventilation, prone positioning, a fluid conservative approach, and deep sedation with paralysis [3,4,5,6]. Additionally, there are data supporting the use of steroids in early, severe ARDS [7]. Nonetheless, treatment is increasingly hampered by the rapid emergence and spread of antimicrobial resistance among bacterial species, which has resulted in pan-resistant strains untreatable with the current armamentarium of antibiotics [8]. There is a pressing need for new therapies.

Recent investigations have demonstrated that mesenchymal stromal cells (MSCs) have significant therapeutic effects in several experimental models of severe pneumonia and sepsis. MSCs have been shown to have a number of biological properties that lend themselves suitable for the acute inflammatory injury seen in ARDS: (a) immunomodulation of excessive pro-inflammatory responses; (b) secretion of reparative epithelial and endothelial growth factors; (c) augmentation of host defense to infection; and (d) avoidance of inciting a host immune response, which allows for allogeneic administration [9,10,11,12,13,14,15,16,17,18,19,20]. The ability of MSCs to avoid eliciting a host immune response is important in their ability to be utilized in an “off-the-shelf” manner. This is particularly important in conditions such as ARDS since patients are critically ill and there is not sufficient time to isolate and purify new MSCs from culture. Given these pleiotropic effects and promising experimental results, MSCs have been studied in several clinical trials targeting patients with ARDS and severe sepsis [21,22,23,24,25]. These efforts have been accelerated given the recent COVID-19 pandemic, and there are numerous trials ongoing worldwide. However, up until now, results from clinical trials with MSCs in ARDS have produced equivocal results. One of the potential reasons MSCs have not produced the expected results when tested in patients is the limited survival of MSCs in vivo, which has been reported in several experimental and clinical studies. Consequently, it is important to develop strategies to boost the survival of MSCs when given to patients with acute inflammatory diseases such as ARDS and thereby improve the therapeutic effect seen with MSCs [26,27,28,29].

There are several approaches being proposed to augment the therapeutic effect with MSCs including pre-conditioning with hypoxia or inflammatory stimuli as well as engineering MSCs to express certain proteins such as hypoxia-inducible factor-1 alpha (HIF-1α) [30,31,32,33]. However, inflammatory stimuli can be cytotoxic to cells and induce apoptosis, while genetic engineering of cells can lead to unwanted adverse effects related to dysregulated cell proliferation and possibly malignant transformation. A potentially important pathway to consider that has been understudied in MSCs to date is that related to the protease-activated receptors (PARs). PARs are G-protein coupled receptors that are classically activated by coagulation-based serine proteases, as well as other proteases such as metalloproteinases, and regulate a diverse array of cellular processes including cell survival [34,35,36]. There are four known PARs, and most are found on a number of different cell types including endothelial cells, platelets, leukocytes, epithelial cells and fibroblasts. Recent literature has shown that MSCs express PARs as well [37,38,39,40,41]. PARs exhibit a unique mechanism of activation that entails cleavage of the receptor near the N-terminal, extracellular region by a protease, which then releases a self-tethered ligand to bind to the active site causing intracellular signaling [33,34,35,36,42,43]. Importantly, there are several peptides available that can act as pharmacological agonists or antagonists of the PARs, and therefore their activation state can be modified on cells, such as MSCs, prior to administration to patients afflicted with severe lung injury. This represents a unique opportunity to optimize the biological properties of MSCs ex-vivo in order to achieve a more robust clinical effect.

## 2. MSCs—Preclinical Studies in Acute Lung Injury

Why have MSCs, traditionally found in the bone marrow, generated so much interest as a therapy for inflammatory lung conditions such as ARDS? To better understand this, it is useful to review the physiology of MSCs in the bone marrow and some of the initial work with MSCs in models of lung injury.

As illustrated well by Ehninger et al., bone marrow MSCs help maintain the hematopoietic stem cell (HSC) environment through a combination of cell-cell contact and secretion of soluble factors [44]. Some of the factors involved in the interactions between MSCs and HSCs have also been invoked in the protective effect of MSCs in lung injury, such as angiopoietin-1 (Ang-1). Therefore, the supportive function MSCs play in their “normal” role in the bone marrow translates to having a reparative role in acute, inflammatory lung injury.

One of the initial studies that provided direct evidence that bone marrow progenitor cells (BMPCs), such as MSCs, may have a reparative effect in experimental lung injury was published by Yamada et al. [45]. In this report, the authors demonstrated that BMPCs were mobilized into the circulation of mice injured with intrapulmonary endotoxin, and that bone marrow suppression with radiation prior to endotoxin instillation led to greater alveolar destruction with emphysematous changes. The data presented suggested that BMPCs differentiated into lung epithelial and endothelial cells supporting the prevailing concept at the time that BMPCs, including MSCs, had direct regenerative properties in the injured lung. These findings were similar to those published by other groups, which also suggested that bone marrow derived stem cells could differentiate into alveolar epithelial cells [46,47].

However, repeated analyses and further reports demonstrated that the amount of MSC engraftment in the lung following injury was small and not enough to explain the protective effect observed. Since then, the paradigm has shifted to MSCs exerting a predominantly paracrine effect in these experimental lung injury models through the production of soluble factors and, more recently, extracellular vesicles. Several studies have been published in the past 10–15 years that have provided evidence for the role of paracrine factors such as prostaglandin E2 (PGE2), interleukin-1 receptor antagonist (IL-1ra), keratinocyte growth factor (KGF), Ang-1, cathelicidins such as LL-37, and defensins. These factors have been shown to mediate key biological effects of MSCs that are important in their therapeutic efficacy in acute lung injury, namely: (a) suppression of excessive pro-inflammatory responses, (b) support of the lung endothelial-epithelial barrier, and (c) augmentation of host defense against pathogens [9,10,11,12,13,14,15,16,17,18,19]. The pathways involved in the production of these paracrine factors have been shown to be dependent on exposure to inflammatory stimuli and/or cell-cell contact with host immune cells.

As a result of the multi-pronged manner in which MSCs assist in resolving lung damage and mediating lung repair, experimental studies have shown that treatment with MSCs exerts significant protective effects in several different models of acute lung injury. These include reports in which MSCs confer substantial survival benefits in mouse models of pneumonia and sepsis. In these studies, MSC treatment also resulted in a reduction in pro-inflammatory mediators, less lung injury and improved bacterial clearance from the lung [13]. In addition, data from testing MSCs in an ex-vivo, perfused human lung model of pneumonia have also been promising [14]. An earlier study by Lee et al. demonstrated that human MSCs reduced alveolar inflammation and damage when administered as a treatment to ex-vivo, perfused human lungs injured with endotoxin [11]. The authors showed that through the secretion of KGF, MSCs augmented alveolar fluid clearance in injured human lungs, a process which can hasten the resolution of pulmonary edema that is associated with conditions such as ARDS.

## 3. MSCs—Clinical Studies in ARDS

Given the significant, reproducible benefit seen with MSCs in experimental models of acute lung injury due to infection, there has been a growing effort to test MSC products in clinical trials of patients with ARDS. The initial trials were primarily phase I, dose escalation studies aimed to test the safety of MSCs in patients with ARDS and to find an optimal dose range. The results demonstrated that MSCs were well tolerated with no attributable serious adverse events, but the most efficacious dose of MSCs has not been determined yet [21,22,23]. One of the examples of these first efforts is the START trial published by Wilson et al. in 2015 [21]. In this trial led by Dr. Matthay’s group at the University of California, San Francisco, patients with moderate to severe ARDS (based on the Berlin Definition) were enrolled in an open-label, dose-escalation, phase I trial. There were a total of nine patients enrolled with each successive group of three receiving a higher dose of MSCs: 1 million cells/kg, 5 million cells/kg, and 10 million cells/kg. No infusion-related events or serious adverse events were reported in these patients. In terms of efficacy, there was a decline in measurements of lung injury, organ failure and inflammation in all three dose groups, but there were no between group differences detected.

After the initial set of trials showed safety of MSCs, the focus has recently shifted to determining whether MSCs confer therapeutic benefit in patients with ARDS and this has been assessed in randomized trials comparing MSCs vs. placebo. The follow up to the START phase I trial was a phase IIa trial by Matthay et al. in 2019 that compared a single intravenous dose of 10 million MSCs/kg vs. placebo in a ventilated patients with moderate to severe ARDS [24]. While MSCs were judged to be safe again in this population, the 28-day mortality rate in the MSC group was 30% vs. 15% in the placebo group. There was a non-significant trend towards more ventilator-free days in the placebo group and a significant difference of more ICU-free days in the placebo group (*p* = 0.05). Additionally, there was no difference in concentration of the inflammatory cytokines, interleukin-6 and interleukin-8, between the MSC and placebo treated patients. While there are several potential reasons for the lack of efficacy with MSCs in this trial (including the low mortality rate in the placebo group), one of the primary reasons appears to be low viability of MSCs infused into patients with a range of 36–85% provided. Non-viable MSCs, as would be anticipated, do not have therapeutic value as has been demonstrated in prior experimental studies [10].

More recently, with the onset of the COVID-19 pandemic there has been accelerating interest and efforts to study the therapeutic effect of MSCs in patients with severe COVID-19 induced ARDS. Currently, there are nearly 30 ongoing or recently completed trials listed in clinical trials.gov. While we are awaiting the data from the bulk of these trials, there was a publication in early 2021 that showed benefit with umbilical cord-derived MSCs (UC-MSCs) in patients with ARDS from COVID-19 [25]. In this study, the authors enrolled 24 patients with moderate to severe COVID-19 ARDS into either 2 doses of UC-MSCs or placebo (dose of 100 million cells on day 0 and 3). Most patients received standard of care for COVID-19 including systemic steroids and remdesivir. The authors found that treatment with MSCs improved survival from 42% (placebo group) to 91% 28 days after the second infusion (*p* = 0.015). Additionally, only the UC-MSC treated patients showed a significant decline in the levels of inflammatory markers over time. It is important to note that the viability of the MSCs prior to administration was approximately 88–96%, which again highlights the importance of optimizing MSC survival in order to achieve significant clinical signals. This is one of few reports showing a significant benefit of MSC treatment in a clinical trial of patients with lung injury, and the results are encouraging for the field of MSCs and ARDS.

## 4. Difficulties in Translating MSCs from Pre-Clinical Promise to Clinical Benefit

There are several potential explanations for why the promising experimental data with MSCs in ARDS models has not fully translated to producing a robust protective signal in patients. One reason may be the variable survival of MSCs pre-administration, which can significantly impact the therapeutic effect achieved as mentioned earlier [21]. In addition, MSCs are typically cleared within about 48–72 h when introduced into an acute, inflammatory environment, and therefore, their ability to decrease lung injury and mediate lung repair is limited by their transient presence [10]. Ex-vivo strategies to pre-program MSCs towards their reparative phenotype and boost their survival when given to patients may enhance the therapeutic efficiency achieved in clinical settings.

Beyond the survival of MSCs, there are other cell specific factors important to translational efforts that have not been fully elucidated. These include knowing the optimal source of MSCs since they can be isolated from several tissues including bone marrow, adipose tissue, placenta and umbilical cord. There are data suggesting that UC-MSCs are more potent than bone marrow or adipose derived-MSCs, but conclusive findings showing that one source is definitively better are lacking [48]. Another cell-specific factor that needs greater focus is understanding the best culture conditions and media for MSCs prior to harvesting for clinical applications as well as the appropriate passage number beyond which not to exceed. There are reports that hypoxic pre-conditioning of MSCs augments the therapeutic capacity of the cells [31,33], and that higher passage numbers, in general, degrade MSC biological function [49,50,51], but these variables have not been standardized.

When considering non-MSC related issues in translating cell-based therapy to patients with ARDS, there are several more that require further guidance. Among these include the optimal dose of MSCs, number of doses if more than one is warranted, best route (intrapulmonary vs. intravenous), and most appropriate timing to achieve the desired clinical result. Prior studies have utilized different protocols with variations in the dosing, route and timing, but we are still awaiting a more comprehensive investigation into these factors. A full exploration of these topics is beyond the scope of this review, but they remain important and unanswered.

Lastly, given the complexities of human disease it is predictable that the findings seen with MSCs in experimental models of ARDS would not easily translate to clinical ARDS. Since ARDS is a heterogenous syndrome with several different causes, it has many different subphenotypes that may make them more or less amenable to treatment with MSC-based therapy. As has been published recently, these subphenotypes can be distinguished, to some extent, by differences in inflammatory markers [52,53]. It stands to reason that since MSCs are potent immunomodulators, the group with the highest inflammatory markers may be the best to target but which markers to measure and what thresholds to use are unclear. Ultimately, it is critical to identify the subgroup(s) of ARDS patients that would be best treated with MSCs in order to achieve successful outcomes.

## 5. Protease-Activated Receptors

Protease-activated receptors (PARs) are G-protein coupled receptors that are classically activated by coagulation-based serine proteases and regulate a diverse array of cellular processes including cell survival. They are expressed on several different cell types including endothelial cells, platelets, leukocytes, epithelial cells and fibroblasts, and have recently been shown to be expressed by MSCs [34,35,42,43]. Thrombin is a coagulation-based protease generated under inflammatory conditions due to infection and is the primary activator of PAR1. Thrombin activates all 4 of the PARs except PAR2, which is activated by factors VIIa and Xa and consequently results in distinct intracellular signaling responses [34]. Therefore, PAR activation on MSCs is influenced by the coagulation system (which is generally activated during times of infection and inflammation as in ARDS) in the host, but our understanding of the effects of PAR activation on the biological properties of MSCs remains understudied. A recent study published by our group showed that PAR1 is involved in regulating MSC survival and therapeutic capacity under inflammatory conditions [37].

Some PARs can be cleaved at more than one site, and the downstream signaling effects observed with PAR cleavage are determined by the site of cleavage, a finding known as biased agonism. This has been convincingly demonstrated for PAR1, in particular, as published reports have shown that PAR1 is classically cleaved by thrombin at residue 41, but can also be cleaved by activated protein C (APC) at residue 46. Thrombin activation of PAR1 results in G-protein coupled, ERK1/2 signaling, while APC cleavage of PAR1 leads to β-arrestin mediated signaling and activation of Akt [54]. Both the ERK1/2 and Akt pathways are important pro-survival signals within cells. Additionally, PAR1 can be activated by matrix metalloproteinases (MMPs), which have their own distinct cleavage sites. Consequently, PARs can respond to a diverse array of extracellular proteases and relay different intracellular signals in order to modulate cell survival and activity according to the microenvironment of the cell.

In addition to having the ability to be cleaved by different proteases at distinct sites, PARs have the added complexity of being able to homodimerize or heterodimerize with other PARs to affect the resulting intracellular signaling pathway that is activated. One of the more investigated examples of heterodimerization among different PARs is the interaction between PAR1 and PAR2. When thrombin cleaves PAR1 at residue 41, the resulting self-tethered ligand can bind intermolecularly and transactivate PAR2. A paper by Kaneider et al. showed that PAR1 activation transitions from causing vascular damage in early sepsis to being protective in late sepsis [55]. This switch in PAR1 effect was shown to be due, at least in part, to transactivation of PAR2 by PAR1. In addition, there is growing evidence describing co-dependent signaling effects involving PAR1 and PAR3 in a range of experimental systems [41,56,57]. For example, it has been recently demonstrated that PAR1-PAR3 mediate APC-induced cytoprotective effects in neural progenitor cells through sphingosine-1-phosphate receptor 1 dependent activation of Akt [58,59].

## 6. PARs and MSCs

The published literature that describes the effects of PARs on MSC biological functions is sparse. One of the initial publications in this field was by Ho et al. that demonstrated that MMP-1 regulates MSC migratory capacity through its cleavage of PAR1 [39]. This was established with the use of anti-PAR1 antibodies that disrupted MMP-1 interaction with PAR1 and inhibited MSC migration towards glioma conditioned media as assessed by a Boyden chamber assay. These results are similar to what has been described in the tumor metastasis field, since previous studies have shown that MMP-1 derived from fibroblasts can activate PAR1 on breast cancer cells and lead to tumor invasion [60]. Activation of PAR1 can lead to cell motility and migration through the activation of ERK1/2, which has been shown to be a central signaling pathway in this process [61].

Another, more recent article by Chen et al. demonstrated that interleukin-1β (IL-1β) can stimulate the secretion of MMP-1 by MSCs, which can then cleave PAR1 on MSCs and result in cell migration [40]. The authors used a combination of pharmacological inhibitors against IL-1β, MMP-1 and PAR1 to establish this autocrine, pro-migratory pathway for MSCs. The findings from this study are particularly relevant to acute inflammatory conditions such as pneumonia and sepsis since IL-1β is a significant driver of the pathophysiology of the acute phase of infection. It is interesting to consider the results of this study in the context of the prior literature of the role of IL-1β pathway on MSC activity. There have been other reports that have demonstrated that IL-1β elicits a response within MSCs that is beneficial by biasing cells towards an anti-inflammatory, pro-trophic phenotype [62]. In addition, one of the original studies in the field of MSCs and lung injury was by Ortiz et al. that reported IL-1ra secretion by MSCs is an important mediator of their protective effect in bleomycin induced lung injury [19]. Therefore, MSCs may respond to exogenous IL-1β through a range of pleiotropic effects that facilitate tissue repair, while also neutralizing IL-1β with the production of IL-1ra.

While the studies mentioned above focused on the role of the MMP1-PAR1 axis on MSC migration, another publication by Chen et al. reported that thrombin promotes secretion of fibronectin by MSCs via activation of PAR1 and PAR2 [38]. They show data that human MSCs express PAR1 and PAR2 by semi-quantitative RT-PCR, and that thrombin stimulation leads to rapid phosphorylation of ERK1/2, which is the classical pathway activated by thrombin cleavage of PAR1 [34,42,54]. Pharmacological inhibition of both PAR1 and PAR2 significantly attenuated fibronectin secretion as did inhibition of ERK1/2. These results suggest that thrombin cleavage of PAR1 and activation of ERK1/2 are involved in fibronectin secretion by MSCs under in vitro conditions. The finding that inhibition of PAR2 also decreases fibronectin secretion is interesting and suggests that transactivation of PAR2 by PAR1 is involved since thrombin does not directly activate PAR2 [34,35]. While this study is primarily relevant for developing alternative methods for culturing MSCs, it is one of the few reports that describe an effect of thrombin on MSC biological activity.

In addition, a recent paper by Sung et al. investigated the role of PAR-mediated signaling pathways in the biogenesis of UC-MSC-derived extracellular vesicles (EV) [41]. They demonstrated that MSCs expressed PAR1 and PAR3 but not significant amounts of PAR2 or PAR4. Then they showed that thrombin preconditioning of MSCs led to an increase in EV production and the levels of certain cargo proteins including Ang-1 and hepatocyte growth factor (HGF), both of which have been demonstrated to mediate protective effects seen with MSCs [63,64,65,66]. To determine if PAR1 and PAR3 mediated the increase in EV number and protein content seen with thrombin stimulation, the authors used the PAR1 antagonist SCH79797 and a PAR3-specific siRNA. They reported that EV formation and protein content were inhibited more by SCH79797 than by PAR3 siRNA, and that the combination of SCH79797 and PAR3 siRNA was the most effective in decreasing EV formation and protein content. This is another example of potential cooperative signaling between PAR1 and PAR3 as has been shown in neural progenitor cells [58,59]. As the authors point out in their discussion, these findings may provide a rationale to use PAR1 and PAR3 specific agonist peptides to boost the therapeutic efficacy of MSCs by increasing the production of EVs.

**Figure 1:**
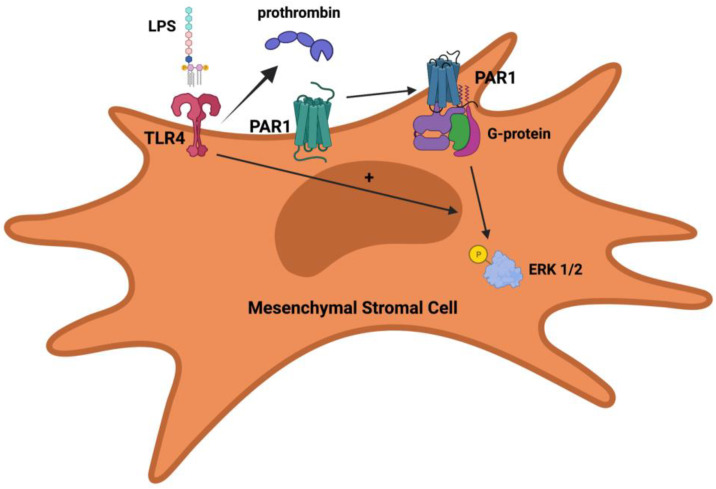
Potential regulation of PAR1 activity in MSCs by TLR4. TLR4 stimulation leads to prothrombin expression by MSCs, which in turn can be converted into thrombin and then subsequently cleave PAR1 on MSCs. PAR1 activation by thrombin leads to G-protein coupled signaling and ERK1/2 phosphorylation. Activation of PAR1 by thrombin and ERK1/2 phosphorylation is dependent on having an intact TLR4 pathway.

With this background, our group recently published an article that examined the role of PAR1 in the therapeutic capacity of MSCs for experimental bacterial pneumonia and sepsis [37]. In this study, we used PAR1-mutant MSCs isolated from corresponding mice generated by Dr. John Griffin’s research group at Scripps Research [67]. The mutation was an R (Arginine) to Q (Glutamine) change at residue 41 near the N-terminal domain of the PAR1 protein, which eliminates the ability of thrombin to cleave PAR1 on cells. As a result, the use of R41Q-PAR1 mutant MSCs allowed us to specifically ascertain the consequences of thrombin cleavage of PAR1 on MSC survival and function. Our findings demonstrated the following: (a) mouse bone-marrow derived MSCs express PAR1; (b) thrombin stimulation led to rapid ERK1/2 phosphorylation in MSCs and a pro-growth, pro-survival effect in MSCs; and (c) R41Q-PAR1 mutant MSCs were more susceptible to cell death when exposed to cytotoxic stimuli in vitro. When tested in vivo, R41Q-PAR1 mutant MSCs exerted no therapeutic effect when administered to mice with severe *E. coli* pneumonia suggesting that PAR1 activation by thrombin is required for MSC conferred protection. In addition, the TLR4 pathway was linked to signaling through PAR1 on MSCs in two ways: (1) stimulation of MSCs with endotoxin led to a low level of prothrombin expression by MSCs; and (2) mutation of the TLR4 receptor on MSCs eliminated signaling through PAR1 on MSC in response to thrombin. The finding that MSCs can express and secrete prothrombin in response to endotoxin is the first description of MSCs producing a coagulation factor to our knowledge, and represents another possible autocrine, protease-based signaling loop that is activated by a pro-inflammatory stimulus. When coupled with the recent findings by Chen et al. [40], a common theme that emerges is that MSCs integrate inflammatory signals in its microenvironment and secrete proteases such as MMP-1 and prothrombin, which can activate PAR1 on its cell surface. PAR1 activation, in turn, can lead to pro-survival and migratory signals that are beneficial to MSCs under these conditions. Furthermore, the dependence of thrombin-PAR1 signaling in MSCs on the presence of an intact TLR4 pathway is analogous to what Kaneider et al. reported in endothelial cells [55]. They showed that LPS stimulation of endothelial cells (presumably through TLR4) induced PAR1-PAR2 complexes to appear on the membrane surface where PAR1 can be cleaved by thrombin and subsequently transactivate PAR2. While the precise interaction between the TLR4 and PAR1 pathways has not been fully delineated in MSCs, the TLR4 pathway may also be controlling trafficking of PAR1 to the membrane and thus regulating the cleavage of PAR1 by thrombin. The Figure 1 below illustrates our current knowledge of the connections between the PAR1 and TLR4 pathways in MSCs based on our recent publication [37]. Understanding how TLR4 regulates PAR1 activity in MSCs more fully may allow for the development of ex-vivo priming techniques that optimize PAR1 activation prior to administration and thereby augment the clinical effect achieved with MSCs in patients.

## 7. Conclusions and Future Directions

MSCs have demonstrated considerable promise as a treatment for acute inflammatory lung injury such as ARDS; however, initial efforts to translate MSC-based therapy have been beset by equivocal results. While there are several potential reasons for the lack of clinically significant data with MSC treatment in patients with ARDS, one of the principal issues may be the variable survival of the MSCs themselves. Since PARs regulate many cell processes including survival, exploiting these pathways could represent a unique approach to improving MSC longevity and thereby their therapeutic efficiency.

The field of how PARs modulate MSC biological and therapeutic activity is in its early stages. Although there are a few studies investigating the role of PAR1 activation (by thrombin and MMP-1) on MSCs, many questions remain unanswered. For instance, how thrombin cleavage of PAR1 affects MSC expression of key immunomodulatory proteins, growth factors, and antimicrobial proteins remains to be determined. Additionally, the effects of cleavage of PAR1 by other proteases, such as activated protein C (APC), have not been fully elucidated. APC has been reported to have vascular protective and anti-inflammatory effects, and one study of APC showed a mortality benefit in patients with sepsis [68]. APC cleaves PAR1 at a different site than thrombin, as mentioned earlier, and this leads to a distinct, β-arrestin dependent signaling response [54]. Investigating the effects of APC on MSC survival and biological may yield additional, important insights.

In addition to further investigating the role of PAR1 on MSCs, there are significant gaps in our understanding of how other PARs can control MSC behavior. MSCs have been shown to express PAR2 and PAR3 by other groups [38,41]. PAR2 is unique in that it is the only PAR that is not directly cleaved by thrombin. It is classically activated by proteases such as trypsin and tissue factor complexes with coagulation factor VIIa and VIIa-Xa [69,70]. Additionally, it can also be transactivated by PAR1′s self-tethered ligand (after thrombin cleavage) upon formation of a PAR1-PAR2 heterodimer as described earlier. The mechanism of PAR2 activation affects the resultant signaling cascade activated within cells and the ultimate biological effect. For example, a study by Badeanlou et al. demonstrated that tissue factor-VIIa activation of PAR2 within adipocytes (a cell type that MSCs differentiate into) leads to suppression of the pro-survival signaling protein Akt and an increase in the release of pro-inflammatory mediators [71]. In contrast, as mentioned, transactivation of PAR2 by PAR1 has been shown to be protective in a sepsis model with beneficial effects on vascular endothelial cells [55]. Therefore, the role of PAR2 in infection and inflammation is complex and likely a result of the balance between these disparate effects. However, there is little published to help us understand how PAR2 modulates MSC biological activity. With the use of pharmacological agents and genetically mutated PAR2 mice, new knowledge can hopefully be obtained that sheds light on this important pathway and how to utilize it to maximize the clinical effect achieved with MSC-based therapies.

Future studies may also need to consider the roles of PAR3 and PAR4 since they can be directly activated by thrombin and can heterodimerize with PAR1 [34,35]. Sung et al. reported that UC-MSCs express PAR3, which is consistent with our own unpublished data [41]. They showed that in thrombin stimulated MSCs, siRNA knockdown of PAR3 independently reduced EV formation and protein content and synergized with PAR1 antagonism to nearly completely eliminate the thrombin mediated increase in EV formation and protein content. This finding of possible cooperative signaling between PAR1 and PAR3 in MSCs is similar to what has been reported in other studies involving neural progenitor cells, as mentioned previously [58,59]. PAR1-PAR3 heterodimerization may mediate signaling in MSCs in response to thrombin or APC, and this may have effects that boost MSC survival and function. In terms of PAR4, additional studies will need to be undertaken to determine if MSCs express PAR4 under basal or stimulated conditions since initial reports have suggested a lack of PAR4 expression.

Collectively, PARs are complex, integral receptors that allow cells to respond to inflammatory, protease-mediated signals in their microenvironment. The role of PARs on MSCs has been minimally studied to date, but initial studies have shown that PAR1, and possibly PAR3, activation is necessary for MSCs to exert their protective effect. Importantly, PAR activation on cells can be readily modified with peptides and other compounds. In fact, a PAR1 antagonist, vorapaxar, has already been studied in several clinical trials targeting patients with cardiovascular disease and was approved by the US FDA for the prevention of thrombotic cardiovascular events in those with a history of myocardial infarction or peripheral artery disease [72]. Additionally, a modified APC, 3K3A-APC, has been shown to have promising results in patients with ischemic stroke when used in combination with thrombolysis or thrombectomy [73]. These findings illustrate that targeting PARs is a clinically feasible approach, and there will likely be several potential methods to modulate PAR activity on MSCs and thereby enhance the therapeutic benefit achieved with MSCs in ARDS.

## Data Availability

Not applicable.
